# Fairness Expectations and Altruistic Sharing in 15-Month-Old Human Infants

**DOI:** 10.1371/journal.pone.0023223

**Published:** 2011-10-07

**Authors:** Marco F. H. Schmidt, Jessica A. Sommerville

**Affiliations:** 1 Department of Developmental and Comparative Psychology, Max Planck Institute for Evolutionary Anthropology, Leipzig, Germany; 2 Department of Psychology and Institute for Learning and Brain Sciences, University of Washington, Seattle, Washington, United States of America; University of Maribor, Slovenia

## Abstract

Human cooperation is a key driving force behind the evolutionary success of our hominin lineage. At the proximate level, biologists and social scientists have identified other-regarding preferences – such as fairness based on egalitarian motives, and altruism – as likely candidates for fostering large-scale cooperation. A critical question concerns the ontogenetic origins of these constituents of cooperative behavior, as well as whether they emerge independently or in an interrelated fashion. The answer to this question will shed light on the interdisciplinary debate regarding the significance of such preferences for explaining how humans become such cooperative beings. We investigated 15-month-old infants' sensitivity to fairness, and their altruistic behavior, assessed via infants' reactions to a third-party resource distribution task, and via a sharing task. Our results challenge current models of the development of fairness and altruism in two ways. First, in contrast to past work suggesting that fairness and altruism may not emerge until early to mid-childhood, 15-month-old infants are sensitive to fairness and can engage in altruistic sharing. Second, infants' degree of sensitivity to fairness as a third-party observer was related to whether they shared toys altruistically or selfishly, indicating that moral evaluations and prosocial behavior are heavily interconnected from early in development. Our results present the first evidence that the roots of a basic sense of fairness and altruism can be found in infancy, and that these other-regarding preferences develop in a parallel and interwoven fashion. These findings support arguments for an evolutionary basis – most likely in dialectical manner including both biological and cultural mechanisms – of human egalitarianism given the rapidly developing nature of other-regarding preferences and their role in the evolution of human-specific forms of cooperation. Future work of this kind will help determine to what extent uniquely human sociality and morality depend on other-regarding preferences emerging early in life.

## Introduction

Since Darwin, the evolutionary emergence and stability of human cooperation – which presents an outlier in the animal kingdom in terms of its scale – has puzzled biologists and social scientists [1]–[3]. This is due to the paradoxical nature of cooperative activities: they are frequently costly to the individual without yielding any direct benefits. Traditionally, natural selection is assumed to favor competition among conspecifics [1], [4], or, even more fundamentally, between alleles [3], [5], but the fact that virtually all human societies are based on cooperation (often among genetically unrelated individuals) has led researchers to identify mechanisms that allowed cooperation to emerge and persist. Nowak [6] proposed five such mechanisms: kin selection, direct reciprocity, indirect reciprocity (based on reputation), network reciprocity [7], [8], and group selection. Further mechanisms that have been suggested to enforce cooperation are punishment including peer- and pool-punishment [9]–[15], reward [16]–[18], and policing [19].

In addition to recognizing ultimate mechanisms that explain why and under which conditions cooperative behaviors are adaptive, a critical charge in building a scientific understanding of human cooperative tendencies is identifying psychological dispositions and traits that enable the operation of such mechanisms in the first place. As such, empirical research using psychological methods is very important for understanding how humans become such cooperative beings over the course of ontogeny. Recently, a range of prosocial dispositional attitudes or “other-regarding preferences” have been identified and promoted as likely candidates to explain why human cooperation has been maintained and developed to a large scale [20]–[23]. Among these other-regarding preferences are fairness (based on egalitarian motives, e.g., a propensity to share resources equally) and altruism (an act costly to oneself and at the same time beneficial to a recipient). Theoretically, these two constructs are interrelated: both require a concern for others, and at times, a willingness or ability to engage in personal sacrifice [22], [24].

With respect to fairness, several studies in experimental economics using bargaining games suggest that adults consider fairness issues in their decision making [25], [26], and that their egalitarian motives even lead them to produce altruistic acts, such as punishing cheaters (who do not contribute to a common pool) or restoring fairness by redistributing others' incomes at a cost to themselves [20], [27]. Neuro-imaging work has confirmed this preference for fairness and found that receiving fair (vs. unfair) offers in bargaining games leads to greater activation in brain reward regions [28], and that aversion to unfair offers is strongly related to amygdala activity indicating an automatic emotional response to unfairness [29]. Regarding altruism, other bargaining games in which adults can choose how much, if any, money to give to another subject (in a one-shot, anonymous setting) showed that over 50% of participants decide to give away their own money and thus perform an altruistic act [30]. These findings suggest that such other-regarding preferences are an entrenched part of human behavior. Yet debate exists as to whether other-regarding preferences are also present in other species [31], [32], or whether they are uniquely human [22], [33].

Past work indicates that other-regarding preferences may emerge fairly late in ontogeny, suggesting the need for a protracted period of socialization. For instance, when required to distribute goods between themselves and a recipient, children do not distribute goods equally until roughly middle childhood [21], [34], [35]. Some studies [34] required children to act against their self-interests to behave in a fair manner (e.g., by donating stickers and thereby decreasing their own share of an endowment). In another study [21] children played allocation games. In the “prosocial game”, children received a candy, and were able to choose whether their anonymous partner received zero candies or one candy. Under these conditions, children younger than seven to eight years of age did not reliably prefer the egalitarian allocation (1∶1). Given limitations in young children's inhibitory control abilities and in the ecological validity of these experiments (e.g., resource distribution devoid of social context), these paradigms, however, may have underestimated young children's abilities. Indeed, evidence suggests that children as young as 3.5 years of age distribute resources fairly when they do not stand to directly benefit from the resource distribution [35], [36]. These findings are consistent with work suggesting that evaluating interactions between other individuals along the dimension of fairness (*third-party fairness*) is distinct from being the victim or agent (*egocentric fairness*) of unfair behavior [37]–[39].

Similarly, experimental evidence suggests that sharing tendencies also develop later in childhood. For example, a recent study demonstrated that it is not until 25 months of age that toddlers voluntarily share resources with an adult who makes her desire explicit [40]. However, this experiment required children to distribute essential resources and to act on complicated apparatuses requiring high attentional and motor demands, which may have limited infants' ability to share resources. Thus, it is possible that infants will be more successful at sharing resources when tested in paradigms that require less complex motoric responses, and involve non-essential resources.

The current experiment investigated the emergence of sensitivity to fairness, and the willingness to share goods altruistically, in 15-month-old infants. Despite the work discussed above, there are several reasons to believe that such other-regarding preferences may emerge early in the course of development. At an evolutionary level such preferences may have been crucial for our hominin ancestors to enable and maintain cooperation in small groups, and later, in larger groups of genetically unrelated individuals, to introduce norms (e.g., how to share spoils after a group hunt) that fostered group cohesion, and to motivate group members to enforce those norms. At a developmental level, infants often evaluate events on the basis of underlying social and physical principles, before they can produce behavior consistent with these principles [41]–[45]. Indeed, a recent study demonstrated that infants may evaluate interactions between agents along morally relevant dimensions [46]. Moreover, there is evidence to suggest that prosocial behaviors, such as empathic concern [47]–[49] and instrumental helping [31], [50] can be detected via both naturalistic observations, and in experimental tasks, during the second year of life.

We investigated 15-month-old infants' sensitivity to third-party fairness using a resource distribution task in a violation-of-expectation (*VOE*) paradigm, and infants' explicit behavioral responses in a *sharing task*, in which they could choose to share resources with an unfamiliar adult altruistically (share a preferred toy), selfishly (share a non-preferred toy), or not at all. By assessing fairness and altruism in infants via both a violation-of-expectancy paradigm and behavioral measures, we sought to empirically validate the hypothesized theoretical interdependence between these two constructs [22], [24], and to understand the underlying nature of infants' potential fairness expectations.

## Results

The study followed a within-subjects design with each infant tested first in the VOE paradigm and subsequently in the sharing task.

### VOE Paradigm

**Figure 1:**
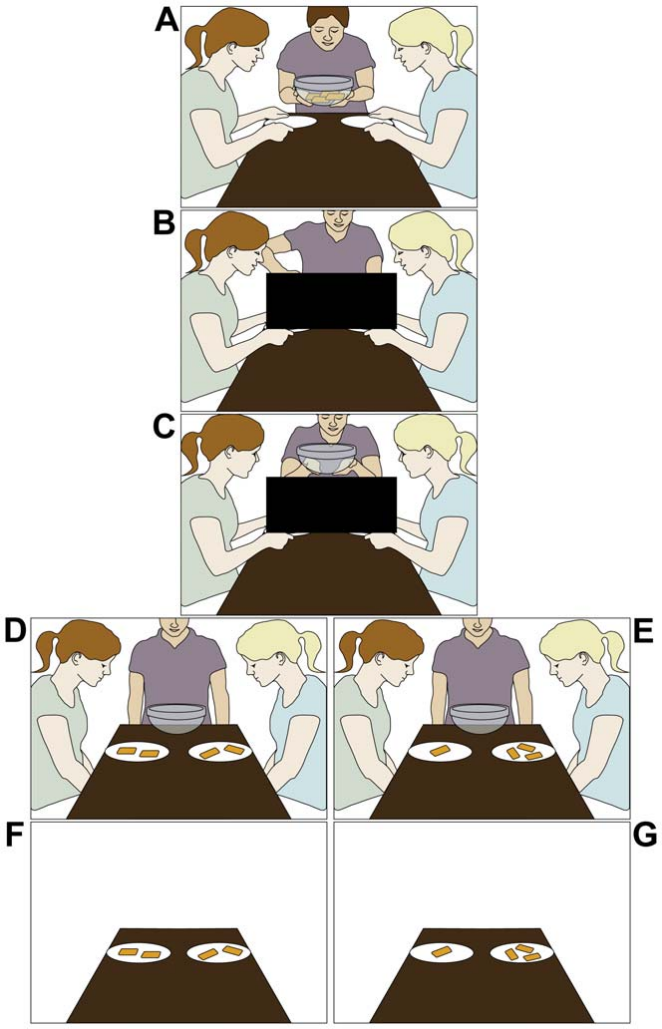
Schematic of the VOE paradigm. In the introductory phase of the crackers movie (milk movie), the distributor greeted the recipients, lifted the bowl of 4 crackers (the pitcher with 10 ounces of milk) while saying “Yummy!” (A). Then, the recipients moved their plates (glasses) toward the distributor asking “Please?”. During the distribution phase (B), the distributor then allocated crackers (milk; exact amount occluded by a black screen) to each recipient via a single movement to each side. The distributor then held up the empty bowl (pitcher) up saying “All gone!” (C). During the test phase, a still frame depicted a fair (D; crackers: 2 crackers each; milk: 5 oz each) and an unfair (E; crackers: 3 crackers vs. 1 cracker; milk: 8 vs. 2 oz) outcome in succession (order counterbalanced), with the actors displaying neutral facial expressions, whereas the post-test phase showed the same displays devoid of a social context, hence symmetrical (F) and asymmetrical (G) outcomes in succession (order counterbalanced).

In the VOE paradigm, infants watched two movies in which an actor allocated continuous (milk) or discrete (crackers) resources to two recipients in a 23-s distribution phase; the outcome of the resource distribution was occluded by a black screen (see Figures 1A–C). In the test phase of each movie, a still frame depicted a fair (Figure 1D) and an unfair (Figure 1E) outcome in succession (order counterbalanced; see Materials and Methods for details), whereas the post-test phase showed the same displays devoid of a social context (i.e., without actors), hence symmetrical (Figure 1F) and asymmetrical (Figure 1G) outcomes in succession (order counterbalanced). Critically, the distribution movement to the recipient receiving more resources than the other recipient (in the unfair test outcome) was 15% longer in duration than the movement to the recipient receiving fewer resources. Thus, besides a social evaluation of the scene (in terms of fairness issues) the paradigm also allowed for a purely “physical evaluation” whose expectations could be diametrically opposed to those of the social one (e.g., an unfair outcome would not necessarily violate any expectations if one solely focuses on the physical aspects of the scene).

Since preliminary analyses yielded no effect of movie type (crackers, milk) on infants' looking to test and post-test outcomes, the data were collapsed across movie type. All statistical tests were performed two-tailed. Analyses focused on looking times collapsed across both movies (*n* = 28), or for one movie (*n* = 19) for those infants that only provided data for one movie, if not stated otherwise. When applying more liberal inclusion criteria (no minimum-look; see Methods for details), *n* = 37 infants provide looking times for both movies (and *n* = 10 for one movie), and the pattern of results (for both sets of analyses) remains the same.

**Figure 2:**
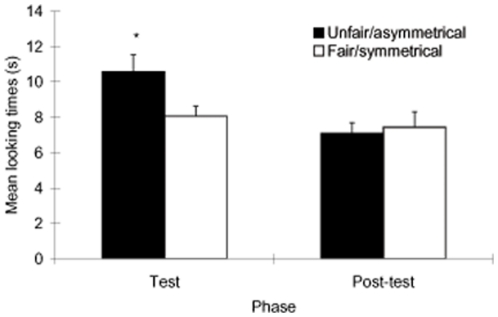
Mean looking times (s) of infants to test and post-test trials collapsed across movie type.

Infants' mean looking times to the test and post-test outcomes in the VOE paradigm are depicted in Figure 2. Infants' expectations in the VOE paradigm were assessed by computing a repeated measures analysis of variance (ANOVA) on averaged looking times with phase (test vs. post-test) and trial-type (fair/symmetrical vs. unfair/asymmetrical outcome) as within-subjects factors. This analysis yielded a significant main effect of phase, *F*(1, 46) = 12.52, *p*<.005, η_p_^2^ *= *.21, and a significant interaction of phase and trial-type, *F*(1, 46) = 4.68, *p*<.05, η_p_^2^ = .09. Planned comparisons revealed that infants looked significantly longer to the unfair (*M* = 10.57 s, *SD* = 6.48) versus the fair outcome (*M* = 8.07 s, *SD* = 3.77) in the test phase, *t*(46) = 2.50, *p* = .02, Cohen's *d* = 0.47 suggesting that these events violated infants' expectations of third-party fairness. In contrast, infants' attention to the asymmetrical (*M* = 7.10 s, *SD* = 4.03) and symmetrical (*M* = 7.44 s, *SD* = 5.96) outcomes in the post-test phase did not differ, *t*(46) = -0.42, *p* = .68, suggesting that infants had no baseline preference for asymmetrical outcomes over symmetrical outcomes.

Infants' attention to the 23-s distribution phase during the first (*M* = 21.21 s, *SD* = 1.43) and second movie (*M* = 21.11 s, *SD* = 1.50), did not differ, *t*(27) = 0.29, *p* = .77, ruling out the possibility that a decline in attention over the course of the experiment led to differential findings across the test and post-test phases. Moreover, there was no difference in infants' attention to the last test trial (*M* = 8.03 s, *SD* = 4.76) and the first post-test trial (*M* = 7.78 s, *SD* = 5.10), *t*(46) = 0.44, *p* = .66, indicating that a failure to find differences in looking to the symmetrical and asymmetrical outcomes could not have arisen from lack of interest in the post-test events.

### Sharing Task and its Interrelations With the VOE Paradigm

**Figure 3:**
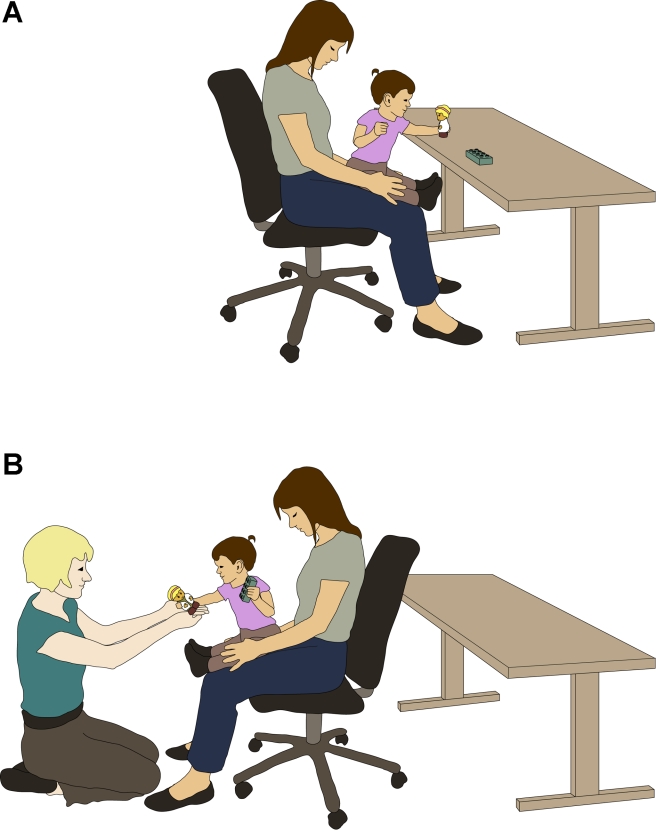
Schematic of the sharing task. In the preference phase (A), the infant chose one of the two toys (only one reachable at a time) - her preferred toy. After the infant had taken one toy, the familiar experimenter gave the infant the other (non-preferred) toy (not depicted here). In the request phase (B), an unfamiliar experimenter asked for a toy while looking directly at the infant. Here, the infant shares her preferred toy (“altruistic sharing”).

Two experimenters (one familiar and one unfamiliar) conducted the sharing task. Two toys were placed on the wooden table 54 cm apart (position counterbalanced). In the preference phase (Figure 3A), infants chose one of the two toys (henceforth labeled the preferred toy). Then, the familiar experimenter gave infants the second (non-preferred) toy. In the request phase (Figure 3B), a second, unfamiliar experimenter (who sat in front of the infant) looked directly at the infant and asked her for a toy (alternating between “Can I have one?” and “Can I have one, please?”) every five seconds for up to 25 seconds. Nine infants were excluded because of a procedural error (*n* = 1), technical error (*n* = 2), missing preference for one of the toys (*n* = 2), or fussiness before/during the preference phase (*n* = 4). Twenty-six out of 38 infants (68%) shared one of the toys: 12 infants shared the preferred toy (32%; “altruistic sharers”), 14 infants shared the non-preferred toy (37%; “selfish sharers”), and 12 infants did not respond at all (32%; non-responders).

Regarding a potential relation between infants VOE performance and their sharing status, it is important to note that if one assumes that infants merely used a formula in the VOE paradigm (e.g., a 15% time difference leads to a 15% difference ratio in outcomes), we would expect no interrelation between infants' VOE preference and their morally relevant sharing behavior, since moral issues of fairness in the VOE paradigm would be irrelevant. To examine the relation between infants' sharing behavior and their VOE performance, we performed two sets of analyses. In the first analysis, we contrasted altruistic sharers and selfish sharers/non-responders. This analysis assumes that infants who shared the preferred toy were motivated by altruistic concerns, whereas those who shared the non-preferred toy, or did not respond at all, were motivated by selfish concerns. Ninety-two percent of altruistic sharers looked longer to the unfair outcome (paired sign test, *p* = .006), whereas 62% of the group of selfish sharers and non-responders looked longer to the fair outcome (paired sign test, *p* = .33; Fisher's exact test, *p* = .004; see Table 1). This association between infants' sharing status and test preference did not arise due to group differences in preferences for symmetrical versus asymmetrical outcomes (Fisher's exact test, *p* = .49): neither altruistic (*p* = .77) nor selfish sharers/non-responders (*p* = .56) differed in their looking to the post-test outcomes. Hence, this tight association between the two tasks validates the VOE paradigm by revealing a relation between variability in third-party social evaluation and “first-party morality” (altruistic vs. selfish sharing), leading to a meaningful dichotomy: altruistic sharers paid attention to fairness issues in the VOE paradigm, whereas selfish sharers/non-responders as a group were not concerned with moral aspects of the VOE scene. Furthermore, these results strongly suggest that the interrelation between the two tasks is based on other-regard, and support the argument that infants were not merely paying more attention to the unfair outcomes because of asymmetry (vs. symmetry in fair outcomes), as this assumption would lead to the theoretically implausible conclusion that altruistic sharers prefer asymmetry, and selfish sharers/non-responders do not.

**Table 1:** Contingency table showing the relation between infants' VOE preference and their sharing status.

		VOE preference		
		Unfair	Fair	Total
**Sharing status**	Altruistic	11	1	12
	Selfish/no response	10	16	26
**Total**		21	17	38

The above analysis assumes that both selfish sharers and non-responders were motivated by the same factor: a reluctance to share the preferred toy. However, it is alternately possible that non-responders were comprised of a heterogeneous group whose performance on the sharing task was governed by factors ancillary to selfish or altruistic concerns. Indeed, there are multiple reasons that infants may fail to respond in our task: because they do not understand the experimenter's request, because they are distracted or inattentive, because they are struggling to decide which toy to select under the allotted time constraints, and/or because they suffer from stranger anxiety.

To investigate whether non-responders differed from responders (i.e., altruistic and selfish sharers) on at least one of the dimensions listed above, we coded all infants for behaviors indicative of stranger anxiety [51]–[53] during the request phase of the sharing task. This dimension was selected for two reasons. First, stranger anxiety is a normal affective response or a form of distress that the majority of infants show during their ontogeny that is orthogonal to moral concerns or motivations [51], [53], and that peaks between 12 to 15 months [51], [54]. Second, stranger anxiety results in a range of identifiable behaviors that could be readily and objectively coded from videotape (concerned/fearful facial expressions, avoiding looking at the requestor, crying, looks to the parent; [51]–[53]). Two non-responding infants could not be offline coded due to technical error; the results would remain the same were these two infants included in the analysis. A Mann-Whitney *U* test on sum stranger anxiety scores (0–4) revealed that non-responders showed more stranger anxiety (*Mdn* = 1.0) than responders (*Mdn* = 0), *U* = 38, *p*<.001. Individual-level analyses (based on 2×2 contingency tables) confirmed this pattern: Seventy percent of non-responders avoided looking at the requestor at least once, versus only 12% of responders (Fisher's exact test, *p* = .001), and 40% of non-responders (vs. 0% of responders) showed concerned/fearful facial expressions (*p* = .004). These findings strongly support the claim that non-responders should be treated as a separate group, as their behavior on the sharing task may be governed by stranger anxiety independent of either selfish or altruistic concerns. Indeed, the non-responders had no preference for either outcome in the VOE paradigm (67% preferred the unfair outcome, 33% the fair outcome; paired sign test, *p* = .39).

Thus, in our second analysis we directly contrasted altruistic sharers' with selfish sharers' VOE performance. We found a significant association between sharing status and VOE performance (Table 2; Fisher's exact test, *p*<.001), and that 86% of selfish sharers looked longer to the fair outcome (paired sign test, *p* = .013). Again, this association was not due to group differences in preferences for symmetrical versus asymmetrical outcomes (Fisher's exact test, *p* = .23), and the selfish sharers had no preference for either post-test outcome (*p* = .18). This finding suggests that infants' morally relevant own behavior (altruistic vs. selfish) is tightly linked to their third-party evaluation of morally relevant situations: altruists pay attention to normative (moral) issues of fairness, whereas selfish infants are interested in non-moral physical aspects of social interactions.

**Table 2:** Contingency table showing the altruistic and selfish sharers' VOE preference.

		VOE preference		
		Unfair	Fair	Total
**Sharing status**	Altruistic	11	1	12
	Selfish	2	12	14
**Total**		13	13	26

## Discussion

The current study provides the first evidence that by at least 15 months of age, human infants possess the rudiments of a sense of fairness in that they expect resources to be allocated equally when observing others (third-party fairness). Furthermore, our findings suggest that sharing non-essential resources (at high or low personal costs) with an unfamiliar adult is also prevalent at this age, which dovetails with natural observations of sharing behavior with familiar adults in young infants [55]. More specifically, even altruistic sharing exists in 15-month-olds: one third of infants shared the toy they preferred despite having the option to share a non-preferred toy (or to not respond at all); and virtually all of these “altruistic sharers” expected third-party fairness when observing a resource allocation situation in our VOE paradigm. Infants who shared a non-preferred toy (“selfish sharers”), however, did not focus on (moral) issues of fairness: they appeared more concerned with whether the test outcomes in the VOE paradigm conformed to the physical parameters of the display, which means that they looked longer at fair outcomes, presumably because these did not correspond to the “physics” of the distribution phase. Critically, the aforementioned findings hold when controlling for fair allocation-inherent perceptual features and cues, such as symmetrical (i.e., equal amounts of resources in two locations) versus asymmetrical displays, and also without controlling for individual differences in sharing behavior (selfish sharers were included in the analyses of the VOE paradigm, and infants, as a group, still showed the effect). Moreover, the interrelation between the two tasks provides supplemental evidence that it is not asymmetry versus symmetry that drives infants' looking behavior.

Were infants merely responding to the test events as violations of non-moral conventions (e.g., that goods are usually divided into equal amounts), there would be no reason to expect a tight interconnection between infants' evaluations of the test events and their prosocial behavior. Thus, we suggest that infants evaluate events along morally relevant dimensions, and not just according to whether such events are consistent or inconsistent with conventional norms. Moreover, the fact that infants' sensitivity to fairness and altruism were interrelated not only lends support to theoretical claims of a close alliance in other-regarding preferences [22], [24], but also suggests that morally relevant evaluations, and behavior of a moral character, develop in a parallel and interwoven fashion. Critically, our findings suggest that the individual differences in fairness sensitivity and altruistic behavior that have been documented in adulthood [37], [38] can be traced back to infancy, suggesting that such individual differences might have deep ontogenetic roots.

Taken together, the present findings strike a new path in social-moral development, because they suggest that in addition to instrumental helping [31], [50], and empathetic concern to others' distress [47]–[49], constituents of fairness understanding and altruistic behavior emerge during the second year of life. Furthermore, this study suggests that besides a general propensity to show concern for others' well-being early in life, egalitarian motives also seem to emerge early in ontogeny, a finding that complements and informs current research emphasizing strong egalitarian motives in adults [20], [27]. Hence, these early emerging other-regarding preferences might be conducive to explaining the evolutionary success of our hominin lineage, since they are considered to be important contributors to cooperation [21]–[23]. Given the early developing nature of such sensitivity and its theoretical relation to the evolution of human-specific forms of cooperation, these findings support the claim that other-regarding preferences have been adaptive in our ancestral small-scale group environments and therefore been transmitted up to the present, most likely via both biological and cultural mechanisms.

With respect to the evolution of cooperation, one mechanism, indirect reciprocity [6], has been suggested to be intimately linked to the evolution of human morality and social norms [56]–[58]. In this vein, our findings may provide an empirical piece of the puzzle of human cooperation, given that early in ontogeny, rudiments of behaviors and skills that may be related to the ultimate mechanism of indirect reciprocity are present. Future work will help elucidate how early moral and prosocial capacities like fairness and altruism interrelate with other skills and behaviors considered important for human cooperation, such as understanding and applying (non-moral) social norms.

## Materials and Methods

### Ethics Statement

The treatment of participants in this paper was in accordance with the ethical standards of the American Psychological Association. The subjects' parents provided written informed consent, and the study was approved by the Institutional Review Board at the University of Washington, Seattle, WA (Application #24231).

### Participants

Forty-seven healthy full-term 15-month-old infants (*M* = 15 months, 8 days, range  = 14 months, 20 days – 15 months, 28 days; 24 girls) participated in the study, recruited from urban and surrounding areas of a mid-size city in the USA. Fifteen additional infants were excluded from the study due to fussiness (*n* = 5), parental interference (*n* = 2), failure to meet the minimum-look criteria for inclusion (*n* = 7; see below), or experimenter error (*n* = 1). Parents provided written informed consent. Each infant received a small present for their participation in the study.

### Apparatus and Materials

#### VOE Paradigm

Movies were recorded with three female actors (a distributor and two recipients), and presented on a 21-inch television monitor. The props used in the movies were four Graham crackers, white plates, a transparent bowl (crackers movie), and milk, transparent glasses (volume of 10 oz.), and a transparent glass pitcher (milk movie), respectively.

#### Sharing Task

A green Lego brick (4 cm width, 7 cm length) and a female doctor toy (4 cm width, 8 cm height) were used as resources, and a wooden table (38×92 cm) served as the location from which infants could choose one toy.

### Stimuli and Procedure

#### VOE Paradigm

Infants sat on their parent's lap (80 cm from the display). Parents were instructed to remain silent, and to close their eyes during the experiment. The movies consisted of an initial greeting by a (fourth) female actor (to attract infants' attention), a familiarization phase (including an introductory phase and a 23-s distribution phase), two test trials (a fair and unfair outcome), and two post-test trials (a symmetrical and asymmetrical outcome). Movie order, test outcome shown first, first distribution side, and location (right, left) of the unfair/asymmetrical outcome were counterbalanced across infants.

#### Sharing task

The procedure of the sharing task is outlined in the Results section.

### Coding and Reliability

All sessions were coded on-line, recorded and additionally coded from videotape by a second independent observer.

#### VOE Paradigm

The minimum-look criterion (accumulated) to the distribution phase was 18.4 s (80% of 23 s). Infants' looking to the test and post-test trials was timed on-line until they looked away for 1 consecutive second (maximum trial length: 30 s; minimum-look criterion: 2 s). The second independent observer coded all subjects for reliability (interobserver agreement: 95%).

#### Sharing Task

The secondary observer coded which toy infants chose, whether infants shared a toy or not, which toy (preferred vs. non-preferred) infants handed the second unfamiliar experimenter (interobserver agreement: 100%). The secondary observer additionally coded subjects for behaviors indicative of stranger anxiety in the request phase (concerned/fearful facial expressions, avoiding looking at the requestor, crying, looks to the parent; interobserver agreement: 94%).
